# Annular ligament reconstruction by suture anchor for treatment of radial head dislocation in children

**DOI:** 10.1186/s12891-015-0642-y

**Published:** 2015-08-05

**Authors:** Jian Wang, Liang-Dong Jiang, Ai-Yong He, Dai-Rong Wang, Jun Zhu, Run-Shan Duan, Cheng Tao

**Affiliations:** Department of Orthopedics, the Second Xiangya Hospital, Central South University, Middle Renmin Road No.139, Changsha, 410011 People’s Republic of China; Department of Orthopedics, Changsha Central Hospital, Changsha, 410004 People’s Republic of China

**Keywords:** Radial head dislocation, Suture anchor, Annular ligament reconstruction, Broberg and Morrey 100-point scale, Radial nerve function, Functional recovery

## Abstract

**Background:**

We investigated the efficacy of annular ligament reconstruction by suture anchor in the treatment of radial head dislocation (RHD) in children.

**Method:**

A total of 20 RHD children nderwent annular ligament reconstruction surgery using suture anchor. Preoperative and postoperative elbow functions were evaluated according to Broberg and Morrey 100-point scale. Recovery of radial nerve function was assessed using the Chinese Medical Association of Hand Surgery Branch of Upper Limb Functional Assessment Standard. All statistical analyses were performed using SPSS version 17.0 software.

**Results:**

All 20 RHD children who underwent the procedure were followed up for a median duration of 24 months. At the last follow-up, the average Broberg-Morrey score was 94.3, with 12 children (60.0 %) showing excellent outcomes (score range, 95 to 100), 7 children (35.0 %) showing good outcomes (score range, 80 to 94), 1 child (5.0 %) displayed a fair outcome (score range, 60 to 79), and 0 (0 %) poor outcome. A significant difference in the excellent-good rate was observed when the elbow function before surgery was compared to after surgery (*χ*^*2*^ = 5.559, *P* = 0.018). The radial nerve function of the 13 RHD children with radial nerve injury also recovered to normal. Among these 13 RHD children, nine exhibited excellent outcomes, 3 showed good outcomes, 1 displayed a fair outcome, and no patient showed a poor outcome. A significant difference in the excellent-good rate of radial nerve function was also observed when before surgery was compared to after surgery in these RHD children (*χ*^*2*^ = 4.887, *P* = 0.027).

**Conclusion:**

Our results strongly indicated that suture anchor is highly effective for reconstruction of the annular ligament and to promote full functional recovery in RHD children, demonstrating that the procedure is an excellent treatment choice in RHD children.

## Background

Radial head dislocation (RHD) is one of the most common elbow fracturesand is most often involves ligament injury and, to a lesser extent, results from congential factors, chondral thinning and other bone conditions such as osteoarthritis or posttraumatic arthritis [1, 2]. As the radial capitellum distal articular surface is slightly to the rear and not entirely vertical with the long axis of the radius, the annular ligament can easily slide to anterolateral of the radial head when the radial head is in supinator position, which may predispose to a transverse tear in the annular ligament and result in RHD [3–5]. RHD is categorized into isolated RHD, chronic RHD, congenital RHD and traumatic RHD [6–8]. Traumatic RHD involving proximal ulna fracture is rare, but in pediatric population it is often found with significant damage to soft tissues, including annular ligament injury [9]. Further, dislocation of radial head without concomitant ulnar fracture or humeroulnar subluxation is an isolated RHD, which is a rare injury [10]. Approximately, 5-10 % of pediatric patients experience traumatic elbow injury, but despite these substantial numbers, treatment guidelines and prognosis varies widely. To treat isolated RHD, some medical professionals suggest taking no surgical measures, while others recommend using splints after manual reduction. However, if the manipulative reduction fails, re-dislocation occurs or, in case of chronic RHD, the annular ligament must be reconstructed after surgical reduction [11, 12]. Chronic RHD in children is caused by progressive deformity and an unacceptable loss of motion, requiring timely intervention (fixation and reduction), as symptoms rapidly deteriorate [13]. Chronic RHD treatment is controvertial. Conservative treatment of chronic RHD is prone to failure and radial head resection is inevitable for correcting the dysfunction, therefore early diagnosis and timely intervention is critical for effective treatment of chronic RHD [14, 15]. Open reduction and corrective ulnar osteotomy have been the main methods in treatment of chronic RHD, but annular ligament reconstruction has received significant attention as a viable treatment alternative [16, 17].

Annular ligament stabilizes the elbow joint and is a strong band of fibers that encircles the head of the radius and holds it in contact with the radial notch of the ulna [18]. Annular ligament prevents dislocation of radial head and limits its forward, backward and lateral displacement [19, 20]. A ariety of methods, such as palmaris longus tendon graft and suture anchor, have recently been developed for construction of the annular ligament to treat RHD [21, 22]. Suture anchor is a bioabsorbable tiny implant used for osteosynthesis and is an excellent fixation device for reattaching soft tissues, such as tendons and ligaments, to the bone [23]. However, various complications are associated with inserting suture anchors, such as stiffness, avascular necrosis, bone nonunion or malunion, and persistent pain [24–26]. Notably, population-based studies on the performance of suture anchors to reattach the ligaments in elbow dislocations linked with radial head showed poor clinical outcomes [27–29]. The efficacy of suture anchor in children is unclear and very few studies report its use in chronic RHD children, with inconclusive results. Therefore, we performed this present study to investigate the clinical outcome of suture anchor to reconstruct annular ligament for treatment of RHD in children.

## Methods

### Ethics statement

This study was approved by the Medical and Health Research Ethics Regional Committees of Second Xiangya Hospital, Central South University. All study participants signed informed consent documents under the guidance of their parents or legal guardians. The research was conducted according to the principles of the Declaration of Helsinki declaration.

### Subjects

Between January 2011 and January 2013, 20 children with RHD were recruited at the Department of Orthopedics, Second Xiangya Hospital, Central South University. The diagnosis of RHD was based on the assessment by an experienced physician after examining the X-ray results: the central capitulum humeri was offset from the axis of the radial neck and the radial head was displaced out of the joint capsule from the lateral or anterolateral, but the radial head and capitulum humeri were normally developed. For chronic RHD, the diagnosis was based on patient history and X-ray examinations to avoid misdiagnosis. According to Bado’s classification, the fractures can be divided into four types: Type I refers to ulnar fracture accompanied by anterior dislocation of the radial head; Type II refers to ulnar fracture accompanied by posterior dislocation of the radial head; Type III refers to ulnar fracture accompanied by lateral dislocation of the radial head; and Type IV refers to RHD combined with fractures of the ulna and radius [30].

### Surgical procedure

Fresh traumatic dislocation of radial head should be managed immediately by timely intervention, such as fixation and reduction, to achieve closed reduction of injury. If closed reduction in patients cannot be achieved, or in patients with an unstable diaplasis and chronic RHD, or incarceration of annular ligament, open reduction and reconstruction of annular ligament is needed. This study contained 14 patients with chronic RHD and 6 fresh fracture patients with unstable closed reduction. All 20 patients received annular ligament reconstruction by suture anchor. Patients were placed in supine position under intravenous anesthesia or brachial plexus anesthesia. Antibiotics were administered prophylactically 30 min before tourniquet was applied to the arm. The injured limb was positioned on the surgery table with elbow flexion ranging from 30° to 60° and forearm pronated to expose radial head, humeroradial joint and proximal radioulnar joint. The average incision length was 3.5 cm and reached the joint directly. Hematoma in the joint of acute injury cases were evaluated. For old/acute injury, dense fibrous tissue were found in the elbow joint and scar tissue or fibrous tissue metaplasia into cartilage were found in 5 cases. These were resected for easier reduction of radial head. An optimal site at the junction of the joint surface and the bone in ulna was selected as an anchor. The anchor was in the middle of the inferior edge of the radial notch, a cartilage facet at the lateral-upper side of the ulna, which is located by the annular surface of radial head. A small ulnar incision was created and 3.0 mm suture anchor (Smith & Nephew) was inserted. The loaded suture anchor and its attached suture were placed into the shaped hole in the bone for suturing the grafted tendon. The palmaris longus tendon of 4 cm average length was achieved by a small incision in the forearm to wrap the radial collar for reduction of radial head. Finally, the loaded suture anchor and its attached suture were fixed to the ulna. In order to ensure annular ligament reconstruction, flex-extension and pronate-supinate motion were applied to adjust the tendon tension. The remaining anchor suture was used to suture both ends of the tendon.

### Evaluation

Broberg and Morrey is a 100-point rating system and was used to evaluate the elbow function in patients before and after surgery [31]. The evaluation consisted of four sections, with 40 points for motion, 20 points for strength, 5 points for stability and 35 points for pain. The categorical rating was: excellent outcomes range from 95 to 100 points; good outcomes range from 80 to 94 points; fair outcomes range from 60 to 79 points; and poor outcomes are below 60 points. The good or excellent outcome was considered as satisfactory, while fair or poor outcome was unsatisfactory.

Recovery of radial nerve function was assessed by the Chinese Medical Association of Hand Surgery Branch of Upper Limb Functional Assessment Standard (http://lib.gdyqs.cn/UpLoadFiles/Article/2013-10/2013100809473899703.pdf). The categorical rating system was: 13–16 points indicates an excellent outcome; 9–12 points, a good outcome; 5–8 points, a fair outcome; equal or below 4 points, a poor outcome.

### Postsurgical management and follow-up

All the patients were immobilized in plaster at 70° to 90° flexion and rotation of neutral forearm for an average 5 weeks (range, 4 to 6 weeks). Heterotopic ossification was not prevented. Once the plasters were removed, assisted exercises were performed, with the supervision of a professional therapist, to improve the elbow function. Shoulder abduction was avoided to reduceelbow stress when the patients were treated with active flexion. All patients were followed up once a month after the surgery for the first six months and then once every six months. In follow up, the elbow function was assessed using the Broberg and Morrey score system. The range of motion was measured by a therapist with a goniometer and plain radiograph was performed to assessjoint congruency.

### Statistical analysis

The nonparametric Mann–Whitney (Wilcoxon) 2-sample rank sum test was applied for comparison between the preoperative and postoperative groups. Data were expressed as mean ± standard deviation. All statistical analyses were conducted using SPSS 17.0 software (SPSS Inc, Chicago, Illinois, USA). A value of *P* < 0.05 was considered as significant.

## Results

### Patient demographics

As shown in Table 1, this study enrolled 13 males and 7 females, with an average age of 6.7 and the ages ranging from 2 to 13 years. All injuries of the patients were unilateral, including 14 right elbow and 6 left elbow. There were 13 RDH children with radial nerve injury and 7 patients without radial nerve injury. Ten patients were Bado type I injuries, 6 patients with type II, 3 patients were type III, and only one patient showed type IV. Open fracture was found in 9 patients and close fracture was observed in 11 patients. A total of 9 fractures were located in the proximal third of the ulna, 7 in the middle third, and 4 in the distal third. The average time from injury to surgery was 8.1 weeks (1–14 months). All patients were followed up for a median of 24 months (range, 9 to 36 months).

**Table 1:** Clinical characteristics of twenty children with radial head dislocation

No.	Gender	Age (years)	Affected limb	Causes of injury	Bado type	Radial nerve injury	Fracture type	Fracture site	TIS duration (weeks)	Follow-up (months)
1	M	6	R	Fall	I	Y	Open	Middle 1/3	1	21
2	F	8	L	Collision	II	N	Close	Distal 1/3	4	33
3	M	7	R	Traffic accident	III	Y	Open	Proximal 1/3	3	36
4	M	5	L	Fall	IV	Y	Close	Middle 1/3	6	28
5	F	7	R	Collision	II	N	Close	Proximal 1/3	5	30
6	M	6	R	Collision	I	Y	Close	Proximal 1/3	7	32
7	F	9	R	Collision	III	N	Open	Middle 1/3	8	29
8	F	8	R	Fall	I	Y	Close	Proximal 1/3	9	21
9	M	10	L	Fall	II	Y	Close	Distal 1/3	10	18
10	M	12	R	Fall	III	N	Open	Middle 1/3	7	9
11	M	4	R	Fall	I	Y	Close	Proximal 1/3	12	12
12	F	3	R	Traffic accident	I	N	Open	Proximal 1/3	11	16
13	M	13	L	Fall	I	Y	Close	Distal 1/3	14	10
14	M	4	R	Collision	II	Y	Open	Middle 1/3	10	32
15	M	5	R	Fall	II	Y	Close	Proximal 1/3	7	30
16	M	7	R	Fall	I	Y	Close	Middle 1/3	8	25
17	F	6	L	Collision	I	N	Close	Distal 1/3	11	23
18	M	5	R	Fall	I	Y	Open	Middle 1/3	12	21
19	M	7	L	Collision	I	N	Open	Proximal 1/3	9	35
20	F	2	R	Collision	II	Y	Open	Proximal 1/3	8	19

M, male; F, female; R, right; L, left; Y, yes; N, no; TIS duration represents the duration from the injury to the surgery

### Improvement of elbow function

**Fig. 1:**
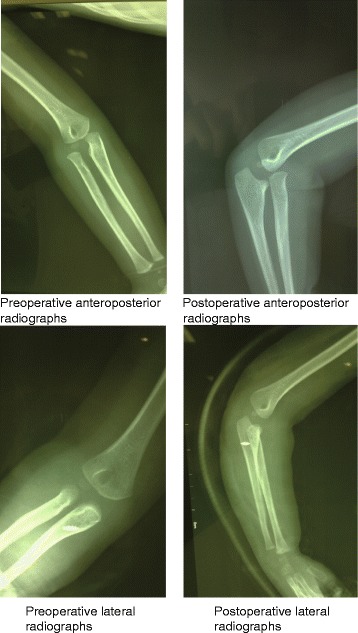
X-ray film of a 10-year-old boy (case 9) with left radial head dislocation before and after treatment with reconstruction of annular ligament by suture anchor. **a** X-ray in the right view of the elbow before surgery; **b** X-ray in the lateral view of the elbow before surgery; **c** X-ray in the right view of elbow after surgery; **d** X-ray in the lateral view of the elbow after surgery

Table 2 shows Broberg-Morrey score of RHD patients before and after surgery. At the last follow-up, the average Broberg-Morrey score was 94.3, with scores ranging between 82 and 100, and 12 patients (60.0 %) showed excellent outcomes (score range, 95 to 100), 7 patients (35.0 %) showed good outcomes (score range, 80 to 94), 1 patient (5.0 %) displayed a fair outcome (score range, 60 to 79), with 0 (0 %) poor outcome (as shown in Table 3). In the excellent-good score range, there was a significant difference between before surgery and after surgery (*χ*^*2*^ = 5.559, *P* = 0.018). All patients underwent post-operative radiographic evaluation. Range of motion was measured with flexion, extension, pronation, and supination. The average flexion and extension were 134.2 ± 3.5° and 8.3 ± 1.4° at the last follow-up, and the average pronation and supination were 85.8 ± 5.3 and 86.4 ± 4.0°, respectively. The postoperative flexion, extension, pronation and supination increased by 12.6°, 2.5°, 16.6°, 24.3°, respectively (Table 4). Follow-up X-ray examinations demonstrated excellent congruity of both the radio capitellar joints and the humeroulnar. X-ray examinations also showed no change in the position of suture anchors and good congruency of the elbow articulation (Fig. 1). All the surgical wounds were healing by first intention. Radial palsy occurred in 1 patient post-operatively, but reverted spontaneously after 3 months. We observed no cases of compartment syndrome, infection, myositis ossificans, posterior interosseous nerve injury, and radial neck fracture.

**Table 2:** The Broberg-Morrey score of elbow function of children with radial head dislocation before and after the annular ligament reconstruction by suture anchor

Time point	Broberg-Morrey score				
	Strength	Pain	Stability	Motion	Total
Preoperative	11.8 ± 2.1	21.4 ± 3.6	2.2 ± 0.8	28.7 ± 3.9	64.3 ± 7.6
Postoperative					
1st month	14.5 ± 3.2	25.6 ± 2.0	3.5 ± 1.1	31.9 ± 3.4	81.2 ± 5.8
3rd month	15.7 ± 2.6	26.8 ± 3.4	3.9 ± 0.8	33.2 ± 5.0	86.9 ± 4.4
6th month	16.4 ± 1.8	27.3 ± 2.7	4.1 ± 0.6	34.5 ± 4.2	88.8 ± 3.2
12th month	17.3 ± 1.5	28.4 ± 1.8	4.3 ± 0.2	36.1 ± 2.9	90.3 ± 1.6
24th month	18.5 ± 0.9	28.9 ± 0.3	4.5 ± 0.1	37.2 ± 1.3	91.2 ± 2.1
36th month	19.1 ± 0.4	29.2 ± 0.3	4.6 ± 0.3	38.1 ± 0.4	94.3 ± 3.5

**Table 3:** The elbow function grade of patients with radial head dislocation on the basis of Broberg-Morrey score before and after surgery

Time point	Elbow function grade			
	Excellent	Good	Fair	Poor
Before surgery	8	5	5	2
Last follow-up	12	7	1	0

**Table 4:** The functional range of motion (°) of the affected elbow before and after the annular ligament reconstruction by suture anchor

Time point	Range of motion (°)			
	Flexion	Extension	Pronation	Supination
Before surgery	121.6 ± 2.7	5.8 ± 0.9	69.2 ± 4.6	62.1 ± 7.2
Last follow-up	134.2 ± 3.5	8.3 ± 1.4	85.8 ± 5.3	86.4 ± 4.0

### Improvement of radial nerve function

The radial nerve function of 13 RHD patients with radial nerve injury recovered to normal. Based on theChinese Medical Association of Hand Surgery Branch of Upper Limb Functional Assessment Standard, the average radial nerve function score was 14 (range, 10 to 16 score) at the last follow-up. As shown in Table 5, nine patients exhibited excellent outcomes, 3 patients with good outcomes, 1 patient with fair outcome, and no patient showed poor outcome. A significant difference in the excellent-good rate was also observed between before surgery and after surgery (*χ*^*2*^ = 4.887, *P* = 0.027).

**Table 5:** Radial nerve function of 13 radial head dislocation patients with radial nerve injury before and after the annular ligament reconstruction by suture anchor

Time point	Elbow function grade			
	Excellent	Good	Fair	Poor
Before surgery	0	7	5	1
Last follow-up	9	3	1	0

## Discussion

RHD can occur under various conditions and RHD in children requires prompt intervention. The main approaches used in children include closed reduction with casting and open reduction and internal fixation. Delayed or inadequate treatment in children may lead to complications such as pain, nonunion, loss of full range of motion or enlargement of the proximal end of the radius [32]. In patients with annular ligament contraction into the joint space or those who suffered re-dislocation caused by an external force, stretching the ligament by manipulative reduction or other non-operative management is very difficult. In this study, chronic RHD children that failed closed reduction via manipulative reduction were enrolled to receive annular ligament reconstruction by suture anchor. Such studies in children are very few and more data is required to understand the best approaches for treatment in children. Therefore, we studied the clinical efficacy of suture anchor for reconstruction of annular ligament in children with RHD. Our study results showed that all patientsshowed excellent recovery with respect to pain and elbow function, suggesting that suture anchors for reconstruction of annular ligament may be highly effective in the treatment of children with chronic RHD or in children who failed closed reduction via manipulative reduction. Earlier studies reported that the material for construction is mostly palmaris longus tendon and triceps aponeurosis, and the procedure is accomplished by drilling tunnels in the ulna, bypassing around the radial neck after threading the construction materials through the bone tunnel [32, 33]. Notably, reconstruction of annular ligament can also be achieved by palmaris longus tendon autograft, which is passed around the radial neck first and then through a bony tunnel to be sutured to itself [17]. However, this surgical method is too complicated and requires ensuring that the longer graft can pass through the narrow ulnar tunnel, which could lead to ulna fracture [34]. In this study, we used suture anchors to reconstruct annular ligament in children with RHD. A significant advantage of our approach is that the annular ligament reconstruction by suture anchorminimizes the risk of overcorrection of ulna in radial head [35]. In addition, the small surgical incision of this technique may reduce stripping of the surrounding tissue and limit surgical trauma, resulting in better recovery of the ligament and wound (http://d.wanfangdata.com.cn/Periodical_xdkf201009024.aspx). The anchors, with two long and absorbable sutures, weave the tendon around the ligament to strengthen the repair of the ligament, preventing re-dislocation of radial head [36]. Previous studies suggested that the use of suture anchors save surgical time and minimize the size of surgical incision, compared to the tendon palmaris longus approach [37, 38]. In addition, the fixation is highly effective in preventing re-dislocation of radial head, with fewer related complications [39, 40]. Consistent with this, our study also demonstrates fewer post-operative complications. The radial head can remain in its original position to allow the growth of fibrous tissue around the sutures, even if the tendon graft fails. Additionally, the sutures cannot prevent the growth of radial head since they are absorbable [25].

On the other hand, repeated dislocation and subluxation can have a huge impact on the articular surface and joint capsule, causing severe complications [41]. Our results indicate that reconstruction of annular ligament is critical for the stability and maintenance of the reduction of radial head because annular ligament was accessed at the incision safely, with shorter operative time, and annular ligament is thick and sturdy with tough internal fixation and low-risk of re-dislocation [6]. Due to the unique function of annular ligament in preventing RHD during rotation of forearms, radius spins inside the annular ligament against the ulna and allows axis rotation [16]. Additionally, without the involvement of annular ligament when spinning, radial head will carry the translational motion, which is associated with the risk of leaving the proximal radio ulnar joint and anterolateral dislocation [42]. Metallic anchors are associated with loosening, migration and chondral injury, and orthopedic surgeons are increasingly using bioabsorbable anchors to solve this issue [25]. Bioabsorbable suture anchors are safe and mechanically stable implants, allowing arthroscopic surgeons to secure soft tissue to the bone in and around the shoulder [25].

The current study has several limitations. First, the small sample size may limit statistical accuracy and universal application. In this study, only 20 children with RHD were recruited and the clinical efficacy of annular ligament reconstruction by suture anchor on RHD might be influenced by random variation. Second, the midterm follow-up and the potential lack of power to detect the complication rates may also influence the short-term and long-term clinical efficacy of annular ligament reconstruction by suture anchor on RHD. Thus, prospective studies and long-term follow-up is needed to further confirm whether suture anchor represents the optimal treatment for isolated old radial head dislocation.

## Conclusion

In conclusion, our study presents preliminary evidence that suture anchor may be effective in reconstructing annular ligament and promote functional recovery in RHD patients. The procedure has the advantage of being a simple operation and is less prone to fracture or re-dislocation. Thus, suture anchors may be an effective and reliable approach for the treatment of children with chronic RHD or in patients with failed closed reduction via manipulative reduction.

## Abbreviations

RHD: Radial head dislocation
UHMWPE: ultra-high–molecular weight polyethylene
